# Effective Control of Non-Native American Mink by Strategic Trapping in a River Catchment in Mainland Britain

**DOI:** 10.1002/jwmg.500

**Published:** 2013-01-24

**Authors:** Jonathan C Reynolds, Suzanne M Richardson, Ben J E Rodgers, Owain R K Rodgers

**Affiliations:** Game & Wildlife Conservation TrustFordingbridge, Hampshire SP6 1EF, UK

**Keywords:** *American mink*, *Arvicola amphibius*, *Arvicola terrestris*, Britain, detectability, invasive species, mink raft, *Neovison vison*, reintroduction, trapping, water vole

## Abstract

The introduction of American mink (*Neovison vison*; hereafter mink) into Europe has had severe impacts on many native wildlife species, including the water vole (*Arvicola amphibius*) in mainland Britain. Although trapping has been widely used to attempt to control mink, managers have little direct evidence of its effect on mink density or distribution, particularly where immigration of mink from nearby areas is inevitable. Such evidence is needed to justify the use of lethal methods in conservation policy. During 2006–2010 we removed mink from the River Monnow Catchment in western Britain, using track-recording rafts to monitor continuously for mink presence, guiding a strategic trapping effort. The area monitored and trapped was increased in stages, from a core sub-catchment with 109 km of water-course in 2006, to a 421-km^2^ catchment with 203 km of water-course in 2009. In each successive sub-catchment, mink detection and capture rates declined rapidly to near-zero levels after trapping began. Detections and captures showed seasonal peaks in every year corresponding to known dispersal periods, but also declined steadily from year to year, with increasing periods in which we did not detect mink. These results suggested that each sub-catchment was cleared of mink within a few months, with subsequent captures attributable to immigration. On average, we detected each mink 5.1 times before capture (daily probability of detection = 0.059 per mink and raft), and trapped them 3.4 days after deploying traps in response. On average, mink entering the area were likely to have been present for less than 13 days before capture. Water voles had been extinct in the Monnow Catchment since the 1980s. During 2006–2008 (starting 6 months after mink trapping commenced), we released 700 captive-bred water voles into the treatment area to re-establish a wild population. Persistence of this population through the 4 years of the project was considered indicative of effective mink control. This study demonstrates that, even in a mainland context, a systematic trapping strategy can have a substantial impact on the density and distribution of a damaging species, in this case allowing the restoration of a native prey species. © 2013 The Wildlife Society

Since its introduction to Europe in the early twentieth century, the American mink (*Neovison vison*; hereafter mink) has had damaging impacts on the conservation status of native prey, generating an interest in lethal methods for its control or eradication (Bonesi and Palazon 2007). Invasive mustelids (Mustelidae) in general have been considered difficult to control or eradicate (King et al. 2009). Evidence that lethal methods can effectively control mink density has largely been limited to small islands (Nordström et al. 2003, Nordström and Korpimäki 2004, Ahola et al. 2006). In the island context, the probability of reinvasion, and therefore the need to repeat removal effort, was related to the degree of isolation from source populations (Bonesi and Palazon 2007), blurring the distinction often made between eradication and control. On larger landmasses, the ambition of permanent eradication is more distant and intermediate steps that address sub-regions may be of limited value and economically untenable (Zabala et al. 2010). Although fur farming (the original source of the wild mink population) is now prohibited by law in Britain (Fur Farming Prohibition Act 2000), in much of Europe fur farming remains a source of reinvasion (Bonesi and Palazon 2007). In this mainland context, the impact of mink removal on mink numbers, and benefits to prey, are unclear. This uncertainty influences long-term policy and resource allocation (Reynolds 2009, Zabala et al. 2010), and can be expected to influence public opinion (Messmer et al. 1999). Therefore, quality evidence is needed to inform and defend policy.

We aimed to develop an evidence-based strategy for control of American mink, especially in a mainland or continental context where the probability of reinvasion is high. Kill-trapping techniques for mink are long established in North America (e.g., Harding 1906), and live-capture methods accommodating concerns about non-targets were developed in Britain by the Ministry of Agriculture, Fisheries, and Food during the 1970s (Bateman 1988). The effectiveness of either kill- or live-trapping to control mink numbers could be inferred only from catch-per-unit-effort or field sign surveys.

In previous work, we found that track-recording mink rafts, which accumulated tracks on a clay-and-sand substrate over periods of 7–14 days, were a more sensitive detector of mink presence than were either field sign surveys or incidental sightings and, if rafts were used systematically, the probability of detecting each mink present was high, even at low population density (Reynolds et al. 2004, 2009; Porteus et al. 2012). This suggested a targeted control strategy in which traps were deployed only where mink were recently detected, economizing on daily trap-checking effort (Porteus et al. 2012). Because evidence of mink occupancy was obtained even when traps were not in use, any control effort organized in this way could potentially document its impact on mink occupancy.

After 2004, the raft-guided approach to mink control was adopted in several contemporary conservation projects throughout Britain, some of which included research elements already reported elsewhere (Harrington et al. 2009, Moorhouse et al. 2009, Bryce et al. 2010, Porteus et al. 2012). Across all such projects, control of trapping effort and documentation of outcomes has tended to be impaired as spatial scale increased because of increased dependence on a volunteer workforce and less systematic methods (Reynolds 2009). We aimed to fill the need for a systematically documented case study to demonstrate the impact of trapping on mink occupancy.

On mainland Britain, the principle driver for American mink control has been conservation of the water vole (*Arvicola amphibius*; formerly *A. terrestris*), which was identified as a Priority Species under the United Kingdom Biodiversity Action Plan following a dramatic decline in abundance and distribution (Department of the Environment 1995, Jefferies 2003). The Species Action Plan target was to restore the pre-1970s range by 2010, by addressing the 2 main factors responsible for water vole decline: habitat loss or degradation, and predation by American mink (Department of the Environment 1995). Our study area was typical of much of lowland Britain in that mink were well established, whereas water voles had been absent for at least 15 years. Although adjacent catchments were likely to act as sources for mink recolonization, no source population of water voles existed nearby. Although reintroduction protocols for water voles were well tested, mink had been found to be inimical to persistence of reintroduced water vole colonies (Moorhouse et al. 2009). Concomitant with our mink control effort in the River Monnow catchment, we reintroduced water voles to part of the catchment, providing both conservation relevance and a considerable challenge. Our study aimed to determine 1) whether our raft-guided mink trapping strategy could successfully control occupancy by mink, despite the lack of isolation from adjacent source populations and 2) whether this impact was sufficient to allow water voles to re-establish.

## STUDY AREA

**Figure 1:**
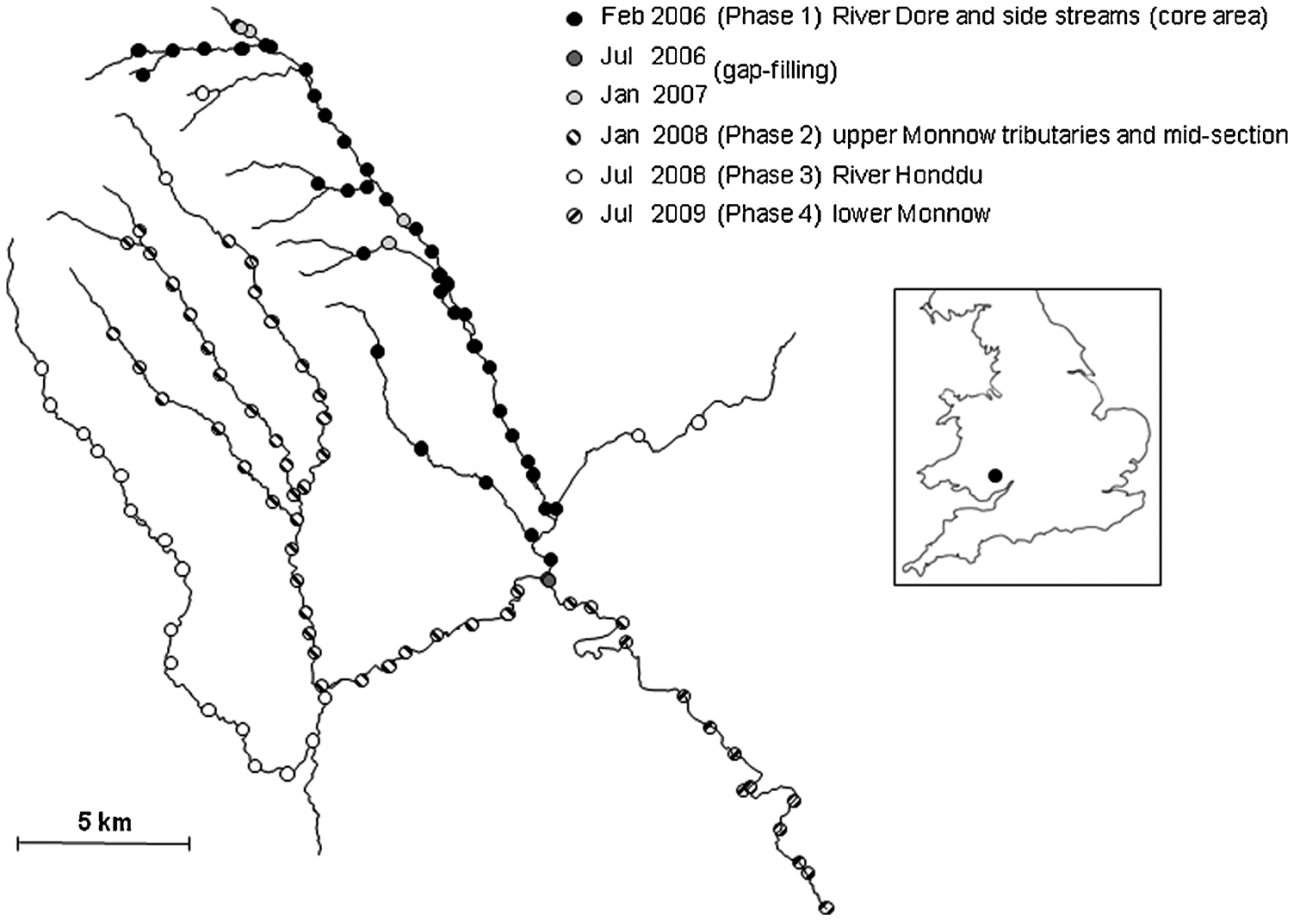
Location (inset) and geography of the River Monnow Catchment, showing the progressive expansion (phases 1–4) of the mink monitoring and trapping (2006–2010).

The River Monnow catchment lies mostly within Herefordshire in western England and drains an area of approximately 420 km^2^ (Fig. 1). The Monnow runs into the larger River Wye at Monmouth. The upper part of the catchment is formed by 5 tributary rivers, of which 4 (Olchon, Upper Monnow, Escley, and Honddu) are high-gradient rain-fed spate rivers flowing over a hard siltstone bedrock and cobble substrate. The River Dore, running over softer, more porous sandstone, has a lower gradient and is more lowland in character, but all 5 tributaries are prone to rapid changes in level and flooding.

Water voles had occurred historically within the Monnow catchment. The last records, both unconfirmed, were in 1982 and 1990 (Herefordshire Biological Records Centre, Hereford; accessed 2006). Our searches at these locations in 2006 found no evidence of water vole presence. An unconfirmed 2002 record suggested a surviving population of water voles on the River Lugg, some 9 km directly to the north-east (40 km by water), but this was subsequently declared extinct (River Lugg Internal Drainage Board 2010).

From the 1970s, riparian habitat in the Monnow catchment was overgrazed by livestock and over-shaded by riverside trees. Our own study was preceded by a fisheries-inspired project from 2003 to 2006, which restored a riverside margin by fencing out livestock and selectively coppicing riverside trees along 90 km of river, and thereby allowed lost riparian vegetation to redevelop (Roberts 2004, 2007). Along the River Dore we judged much of the resulting riparian habitat suitable to support a water vole population, in terms of food plants and substrate suitable for burrowing (Strachan 1998; D. Gow, Derek Gow Consultancy Ltd., personal communication), although it was typically less than 5 m in width and fragmented by stretches of unsuitable habitat. We considered the other 4 tributaries to be unsuitable for water voles throughout most of their length because of the hard bedrock and steep river bed, but they potentially held source populations of mink.

## METHODS

### Mink Control

We conducted this study between 1 March 2006 and 28 February 2010, expanding the area addressed (treatment area) in phases (Fig. 1 and Table 1). In phase 1, we concentrated monitoring and trapping effort in the River Dore and its side streams. We defined this sub-catchment as the core area because control effort was unchanged here throughout the study period and all releases of water vole took place within it (see below). We placed 44 rafts in the core area in March 2006 and checked them all for the first time on 18–20 April. Because of evident seasonal patterns in mink detections and captures, we used this mid-April period in successive survey years for comparison. All fieldwork was conducted by 1 (Mar 2006 to Nov 2007, inclusive) or 2 (Dec 2007 to Feb 2010) fieldworkers. No other mink trapping took place anywhere in the Monnow catchment during the study.

**Table 1:** Control effort and mink presence in the River Monnow catchment during each year of the study between March 2006 and February 2010. The core area refers to the tributary River Dore and its side-streams, which we monitored and trapped throughout the 4-year study. For analysis, years run from March to February

	Year			
	
	1	2	3	4
Control effort				
Area controlled	109 km^2^	109 km^2^	350 km^2^	421 km^2^
River length	63 km	63 km	172 km	203 km
Mink rafts in use	44	48	105	114
Mink killed (entire treatment area)	36	30	26	23
Mink killed/km (entire treatment area)	0.57	0.43	0.15	0.11
Mink killed (core treatment area)	36	27	15	3
Mink presence in core area				
Mink detections per raft and check period (including captures)	22%	8%	4%	1%
Percentage of raft sites at which mink detected during year	95%	84%	70%	53%
Total mink-free days in core area	0 days	98 days	159 days	264 days

We used mink rafts to guide trapping, following previously established protocols (Reynolds et al. 2004, 2009, 2010; Porteus et al. 2012). We deployed rafts at a spacing of approximately 1 per km (average 1.1 km, range 0.14–3.79 km). Initially we checked rafts for mink tracks every 2 weeks as in our earlier protocol, but from 2 October 2006, we intensified this to 1-week check intervals in the mating season (late winter–early spring) and main dispersal period (late summer–autumn) when mink detections were more frequent, to shorten the response time between detection and capture. We identified and recorded all tracks on the tracking medium along with any other field signs (e.g., feces) on the rafts, and used cameras to record tracks if we were unsure about species identification. If we found mink-like tracks, we recorded them as either definitely mink, probably mink, or probably not mink; in all these cases, we set a trap on the same raft. We describe this placement of a trap on a raft in response to mink tracks as a trap deployment. To set a trap, we replaced the tracking medium with an unbaited wire mesh live-catch trap (Rhemo Ltd., New Milton, Hampshire, United Kingdom), which we then checked daily until it caught a mink, or for a pre-determined maximum period (see below). We used live traps because of the risk of catching protected mammal and bird species. We checked traps daily, in line with United Kingdom animal welfare legislation. In each river section, detections were uncommon following the first few weeks of mink removal; subsequent new detections commonly occurred on several adjacent rafts in the same check period. We interpreted this to imply exploratory movement along the river, because home ranges of sedentary mink are generally <4 km of river length (Dunstone 1993). In such situations, we extended our protocol to allow precautionary trap placements on neighboring rafts at either end of the string of rafts, intended to anticipate movement in either direction.

We euthanized each mink on capture, removed the successful trap, and restored the raft to monitoring mode to establish whether or not additional mink remained to be caught. Occasionally, if neighboring rafts or other field evidence already suggested that another mink was present in the vicinity (e.g., if we observed 2 sizes of mink track), we re-set traps immediately; in these cases, we treated continued use of the trap as a fresh deployment in subsequent calculations. Following a capture, other traps already set in response to tracks left by the same mink at other raft sites were potentially redundant if no other mink was present. This situation could not be recognized during use; hence, our protocol was to terminate all trap deployments after 10 days and return the raft to monitoring mode (Porteus et al. 2012). In a few exceptional circumstances (e.g., following an escape from a trap), we continued trap deployments beyond 10 days.

We recorded tracks and other field signs of potential non-target species on or around rafts. This allowed assessment of the risks created by mink trapping for species protected under United Kingdom law, notably otter (*Lutra lutra*), polecat (*Mustela putorius*), water vole, and all waterbirds (European mink [*Mustela lutreola*] does not occur in Britain). From a functional perspective, frequent non-target captures could reduce the availability of traps to catch mink, and confusion between tracks of mink and non-targets could also generate a misleading impression of success or failure. Polecat tracks on rafts were a source of potential confusion with mink tracks. Where doubt over identification occurred, we recorded tracks as described above and set a trap as a precautionary measure. Polecats, stoats (*Mustela erminea*), and non-target bird species were released if caught. Grey squirrels (*Sciurus carolinensis*) were occasionally a nuisance on rafts by digging in the clay medium, and in these cases we trapped and killed them to allow normal function of the raft as a mink detector.

To seek evidence of mink breeding within the treatment area, we classified all mink caught between April 2006 (project start) and 23 October 2008 (*n* = 68) as either juveniles (born within the current year) or adults of breeding age by measuring pulp cavity occlusion in the upper canine teeth (King 1991, Goddard and Reynolds 1993). After dispatching captured mink, we sexed and weighed them, and examined females for evidence of lactation. We removed an upper canine tooth, cut it transversely, and mounted it with the cut surface horizontal. Under a microscope, we then measured tooth width and cavity width in 2 dimensions at right angles, using an eyepiece graticule. We then calculated the average percentage of tooth width occupied by dentine and enamel.

### Water Vole Reintroductions

We released 700 captive-bred water voles (Derek Gow Consultancy, Broadwoodwidger, Devon, United Kingdom) along the main channel of the River Dore between August and September in 2006, 2007, and 2008, following established procedures (D. Gow, personal communication). We transferred voles from handling crates to release boxes provided with straw bedding, shelter, and food. In each release box, we placed sibling groups of up to 6 juveniles, or smaller groups of adults which had been housed together. We spaced release boxes at least 25 m apart along stretches of river bank habitat that we judged to be suitable. Circular holes with a 25-mm diameter in the box sides allowed voles to leave and return; these would have excluded large predators (cat [*Felis domesticus*], fox [*Vulpes vulpes*], gray heron [*Ardea cinerea*], male mink), but not smaller ones (female mink, stoat, weasel [*M. nivalis*]). We provided food daily until voles had vacated the boxes, typically within 3 days.

In 2006, we restricted vole releases (*n* = 300) to the upper half of the river, because by July 2006 this section had no detections of mink, and because we expected the probability of re-invasion by mink to be a function of connectivity. In general, we considered available habitat to be better for water voles in the lower half of the river, with wider river margins and more luxuriant non-woody vegetation; in 2007, we restricted all vole releases (*n* = 360) to the lower half of the river. In 2008, we made further releases (*n* = 40) into gaps in the existing distribution, and at 1 pond 150 m from the main river.

We surveyed the geographical distribution of water voles within the core area once a year in April or early May, when sparse spring vegetation allowed easy observation and positive identification of diagnostic feeding signs, food stores, or feces by fieldworkers wading in the river (Strachan 1998). At this time of year, grazing on the food plant water, dropwort (*Oenanthe crocata*), and larders formed of its stems provided clear evidence of water vole presence. We recorded footprints, runways at the water's edge, and burrows as supportive but not definitive evidence. We searched the entire main channel of the River Dore, and recorded and mapped all field evidence of water voles using a Global Positioning System receiver with a typical accuracy of 10 m.

### Statistical Analysis

Raft checks produced data on naïve site occupancy status, which is the product of 2 unknowns: actual occupancy and detection probability at occupied sites. Actual occupancy is the result of population processes within the catchment, including trapping and immigration. Detection probability is dependent on the number of animals accessing each occupied site, and their individual detectability (defined as the probability of detection per mink and per unit survey effort).

**Figure 2:**
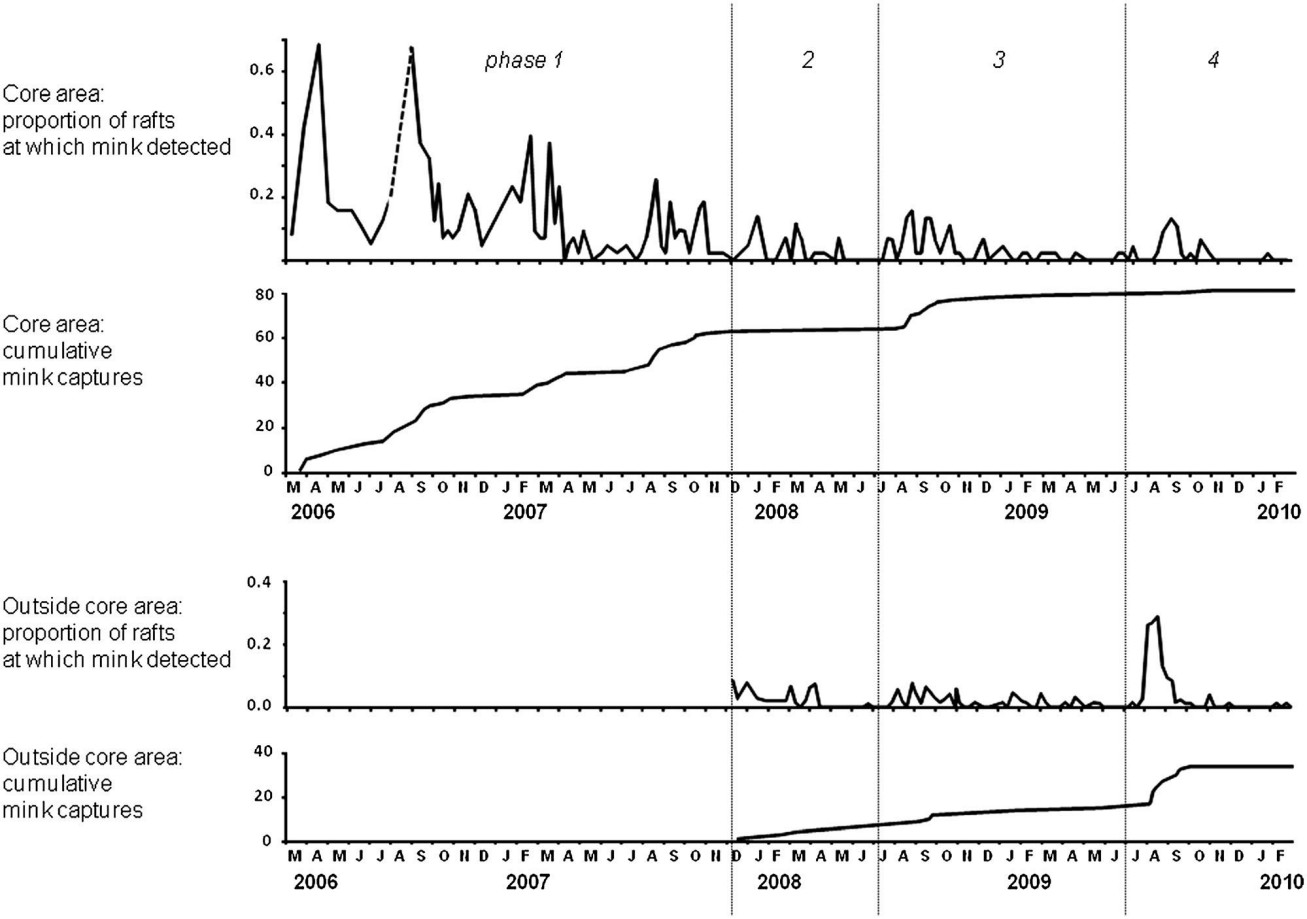
Temporal trends in mink detections and cumulative captures within the core area (upper 2 graphs) and outside the core area (lower 2 graphs) of the River Monnow catchment, during 4 years from March 2006 to February 2010. The upper graph of each pair shows the proportion of rafts at which mink were detected by tracks or by capture in each monitoring period, plotted against the mid date of the period (average period length 9.3 days). Months of the year are indicated on the horizontal axis. Detections include some cases in which the operator expressed uncertainty about identification as mink. The broken line segment in the topmost graph indicates a period in which no monitoring or trapping took place because of lack of available manpower. Vertical dotted lines and numbers 1–4 indicate phases of expansion of the area monitored and trapped. We placed 43–44 rafts within the core area; outside the core area, we placed no rafts in phase 1, 43 in phase 2, 61 in phase 3, and 72 in phase 4. The relatively large peak in detections outside the core area at the start of phase 4 therefore involves 36% of only 11 new rafts.

Without a complex experimental design, changes in occupancy and population size are unavoidably confounded with changes in detectability. Fitting competing statistical models to our data would be useful to establish whether observed seasonal and year-to-year patterns in naïve site occupancy more likely reflect real changes in population size, or are the result of changes in detectability (i.e., to disambiguate these 2 components). However, such models would need to accommodate uncertain detection, site-specific variation in the probability of occupancy, rapid population turnover, both spatial and temporal autocorrelation, and ambiguity between immigration and changes in detectability. They would also have to cope with the fact that rafts equipped with traps are in competition with each other and with rafts in monitoring mode; and that detection leads to a high probability of removal. We do not know of any model framework that can accommodate this demanding scenario. For the present, we therefore use the dynamics of naïve occupancy as the best indicator of impact (Fig. 2; Game & Wildlife Conservation Trust 2012).

We made a point estimate directly from capture data of the rate at which detected mink re-visited rafts. To do this, we assumed that a mink which visited the raft had the same probability of detection whether the raft was in monitoring mode or trapping mode (i.e., no effect of adding the trap), and that this was constant throughout the study. We also assumed that we eventually captured all detected mink. We defined *T* as the number of trap deployments and for each deployment, recorded the number of mink caught per trap per day (*m*; 0 or 1), the number of non-target species caught (*n*), and the day of capture (*c*), counting the day on which the trap was set as Day 0. We then estimated the daily visit rate per mink and raft as: captures/opportunities = [Σ*m*]/[(*T* × *C*) − *n*], where *C* = mean of *c* across all mink caught. We used *C* as the measure of opportunity to catch in place of the total trap-days (1 trap set for 24 hr = 1 trap-day) because after a capture, other traps remaining set in response to tracks left by the same mink would have had no further opportunity to catch it.

An influential parameter in managing predation by mink is the time taken to capture each mink present, especially when reinvasion occurs (Harrington et al. 2009). To obtain an estimate of the response time across all 4 years of our study we added the average check interval to the average number of days *C* until a detected mink was caught, calculated as described above. Again, we have to assume that all mink detected on rafts were subsequently captured.

## RESULTS

### Mink Detections and Captures

**Figure 3:**
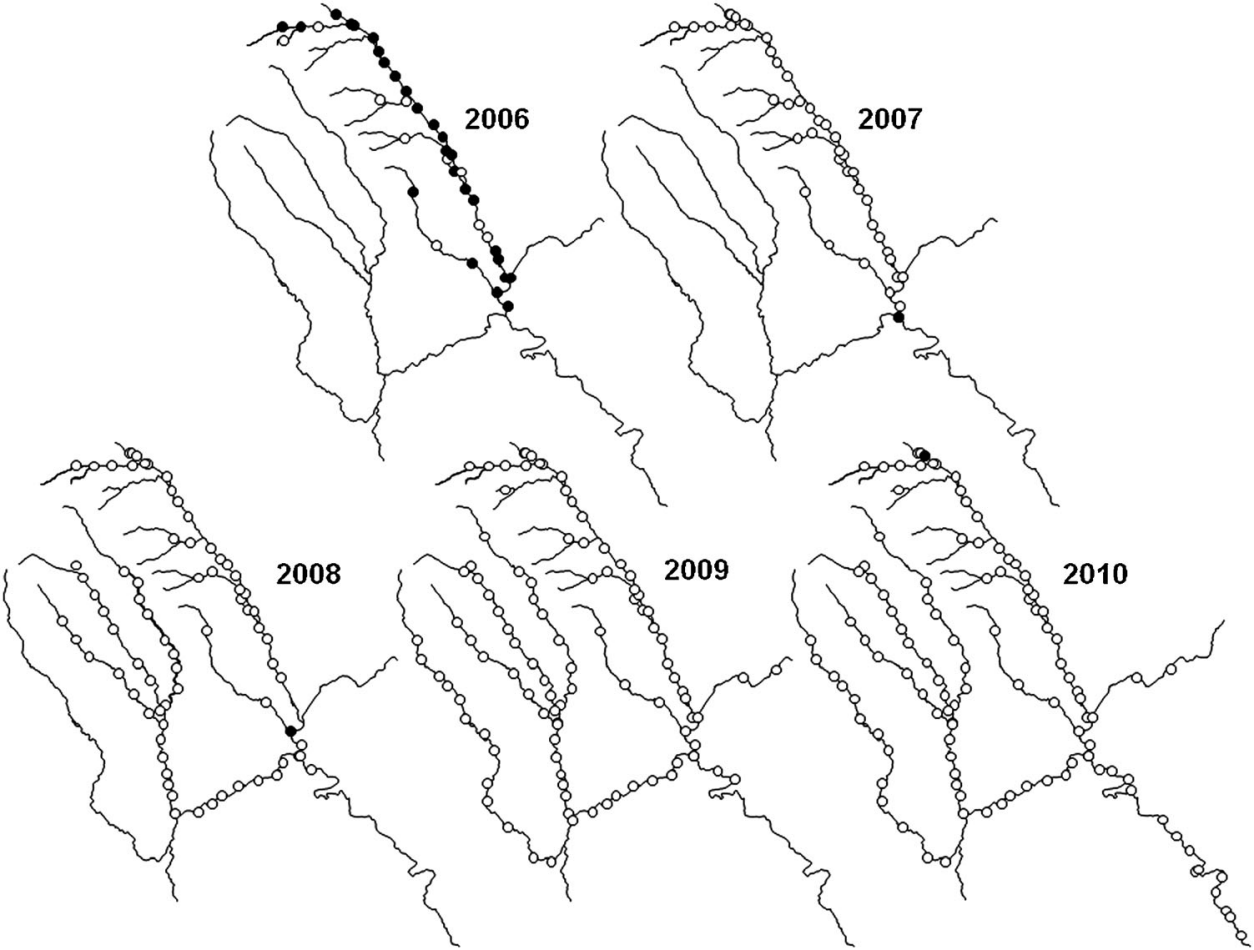
Detections of mink in the River Monnow catchment, during a mid-April check period in each successive year of the study for comparison. Each circle indicates a raft site. White fill indicates a raft in monitoring mode but showing no mink tracks; black fill indicates a mink detection or capture at that raft site. Detections of mink indicated in the maps for 2007, 2008, and 2010 were at the edge of the current raft array. The single mink detection in April 2010 was at a suspected point of entry from the adjacent catchment, and was matched by a capture on the following day.

Across all 4 years, the mean interval between successive checks of rafts in monitoring mode was 9.3 days (range 3–45; mean 12.4 days in 2006, 8.8 days during 2007–2010). Within the core area, the number of mink detections per raft check (combining tracks recorded as definite and probable mink) fell progressively during the first 24 months of the study (Fig. 2 and Table 1). Superimposed on this trend, we found clear seasonal increases in detections (Jan–Mar and mid-Jul–mid-Nov) corresponding to the known mating and main dispersal seasons, which were consistently matched by captures. The use of traps and number of captures decreased in successive years, reflecting fewer detections. Although mink were present somewhere in the core area throughout year 1, the number of check intervals with no mink detections increased year-to-year; in year 4, these totaled 264 days (additional mink-free days could have occurred within check periods in which mink were detected or caught). In April 2006, we found mink throughout the core area. By April 2007, mink were apparently absent throughout the core area (Fig. 3), and almost entirely absent at the same time of year in 2008, 2009, and 2010. In April 2010, we detected mink at only 1 raft site in the entire Monnow catchment, and we caught that mink 2 days later.

Mink detections and captures were clumped geographically. During the first year of mink control, 12% (27/221) of all mink detections and 36% (14/39) of captures occurred on 3/44 rafts within 0.6 km of the confluence of the River Dore with the River Monnow. Even so, mink detections became less widespread with time: among the 44 core area raft sites, the percentage with mink detections in each 12 month period fell consistently through the study from 93% in year 1 to 53% in year 4 (Table 1).

Few mink detections and captures occurred outside the core area except during phase 2 and phase 4 expansions of control effort into the middle and lower sections of the Monnow, which resulted in new clusters of detections and ensuing captures (Figs. 1 and 2). In the first 6 months following each of these expansions (phase 2 and 4), 46% (16/35) and 45% (25/56), respectively, of all mink detections occurred at the new raft sites.

### Trapping Effort and Efficiency

Across all 4 years, we operated traps for 4,123 trap-days. This included traps set in response to tracks classified as definite mink, probably mink, and probably not mink, as well as some supportive deployments on adjacent rafts. Trap use was greatest in year 1 (1,042 trap-days), compared with 666 trap-days in year 4. We caught 115 mink in the 4 years of study. The number of mink caught per year decreased from 36 in year 1 to 23 in year 4, despite the increasing size of the trapped area (Table 1). Across all 4 years, the trapping effort per mink capture averaged 36 trap-days.

**Figure 4:**
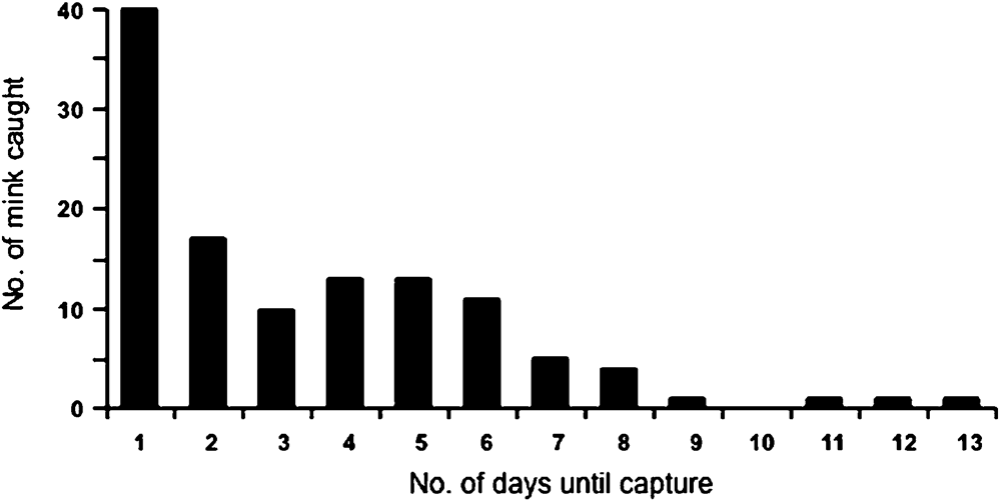
Number of days till capture for all mink caught on the Monnow catchment between March 2006 and February 2010 (*n* = 115). The day we deployed traps in response to tracks on rafts was defined as Day 0 in each case. Mean time to capture was 3.4 days.

We continued individual trap deployments for an average of 6.3 days (range 1–16), but successful deployments caught mink after an average of 3.4 days (range 1–13), with 35% of all captures made on the first day (Fig. 4). Average response time from first detection opportunity to capture was 9.3 + 3.4 = 12.7 days.

We deployed traps in response to tracks categorized by the operator as definite mink or probable mink on 574 occasions among 90 rafts totaling 3,758 trap-days (Table 2). These led to 113 of the 115 mink captures. Twenty-seven non-target animals were caught in the same trap sessions. Thus, the daily probability of capture per trap, for the average mink, is estimated as captures/opportunities in trap-days = 113/[(574 × 3.4) − 27] = 0.059. This implies that detection per mink would be 95% certain given 8 raft-weeks (i.e., the probability of failing to detect for 8 weeks = (1 − 0.057)^8×7^ < 0.05).

**Table 2:** Mink trap deployments in the Monnow catchment, Herefordshire from March 2006 through February 2010, with their duration and mink capture success. We made most deployments only in response to mink-like tracks, and categorized them by the operator's confidence in track identification

Operator's description of track	No. of trap deployments	Total trap-days	Mink captures	Non-target captures
Definite mink	381	2,475	101	15
Probably mink	102	718	3	10
Definite mink on adjacent raft	91	565	9	2
Probably not mink	52	365	2	5
All deployments	626	4,123	115	32

Included in this calculation are 91 supportive trap deployments (565 trap-days) on rafts which had not recorded mink tracks, but where mink tracks had been found on neighboring rafts (Table 2). Nine of these supportive deployments made mink captures, in an average of 2.8 days. Because these traps would have been competing for the same mink with traps placed on rafts with tracks, excluding them from the analysis was not possible, but given their small contribution to either effort or capture success, they can have only a small effect on the estimated re-visit rate. For the same reason, determining whether this tactic of supportive deployments increased the probability of capture was not possible.

Also included in the above calculation are 102 trap deployments (718 trap-days) on rafts showing tracks which the operator classified as mink but with some uncertainty expressed (probably mink). These resulted in 3 mink captures and 10 non-target captures. Excluded from the calculation are 52 precautionary trap deployments (365 trap-days) on rafts which showed tracks similar to mink, but which the operator judged to be probably not mink. These resulted in 5 non-target captures and 2 mink captures (in 1 of the latter cases, definite mink tracks had been recorded on a neighboring raft). Across all trap deployments, non-target captures (including re-captures) comprised 22% of all captures: gray squirrel (*n* = 14), polecat (*n* = 9), brown rat (*Rattus norvegicus*; *n* = 4), stoat (*n* = 2), water rail (*Rallus aquaticus*; *n* = 1), water vole (*n* = 1), and weasel (*n* = 1).

### Evidence of Reproduction Within the Treatment Area

**Figure 5:**
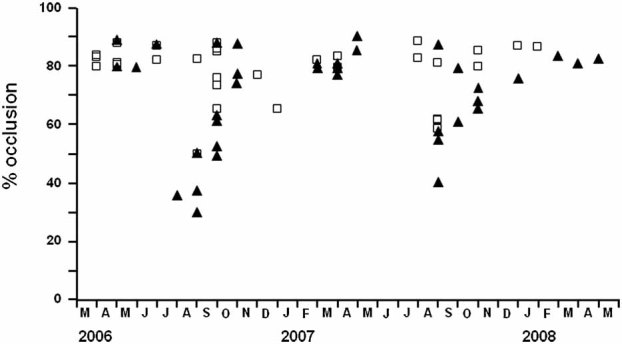
Percentage canine pulp cavity occlusion for male (filled triangles) and female (unfilled squares) mink caught between March 2006 and May 2008 (inclusive) on the Monnow catchment (*n* = 68). Young-of-the-year were clearly distinguishable from adults during July through September. We used percent occlusion <78% as the criterion for classifying mink as young-of-the-year during this period. Months of the year are indicated on the horizontal axis.

The proportion of males among all mink caught was 0.58 (*n* = 115). We found no evidence that this changed during the study: 95% binomial confidence limits on this estimate embraced 0.5 (equal sex ratio) throughout the accumulation of this sample. In each summer, juveniles were clearly distinguishable from adults by canine pulp cavity occlusion, but the 2 classes merged towards the end of September at about 78% occlusion (Fig. 5). For mink caught during October through June, tooth occlusion averaged 82.1 ± SD 4.9. Defining juveniles as those with occlusion of 78% or less, the juvenile:adult ratio was 0.58 in the first 12 months of the study, and 0.64 in the second 12 months.

Of the 49 adult females captured during summer months, only 2 in 2006 (24 Jun, 7 Aug) and 1 in 2007 (14 Aug) showed signs of recent lactation. The earliest date we captured a juvenile was 21 July (in 2006); this was a male weighing 0.85 kg, which would have been about 8 weeks old and likely to be weaned and capable of dispersal (National Research Council Subcommittee on Furbearer Nutrition 1968, Dunstone 1993). Juveniles caught later in the year were likewise judged mature enough to have been dispersing. During autumn, groups consisting of adult female and juvenile mink were taken at nearby rafts, suggesting dispersal as a family group (see Game & Wildlife Conservation Trust 2012). In summary, we found no evidence of young being reared to maturity within the treatment area after trapping had begun.

### Water Vole Persistence

**Figure 6:**
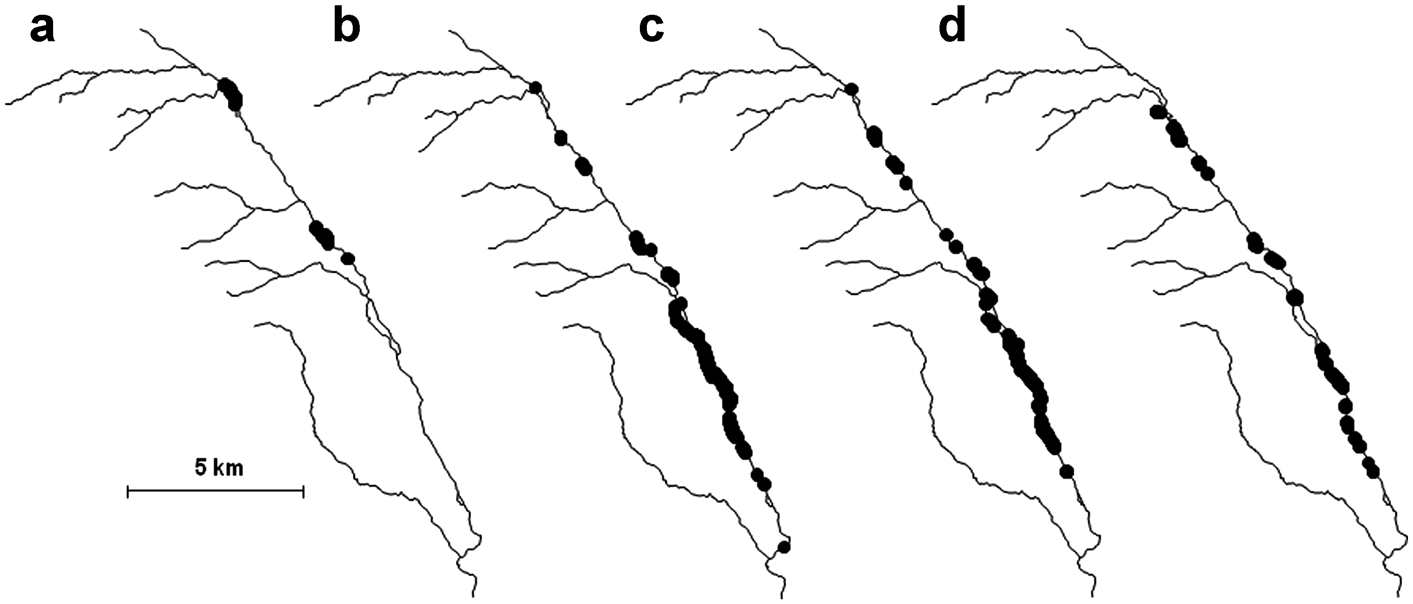
Distribution of water voles within the core area (River Dore) on successive spring field-sign surveys (a) April 2007, (b) April 2008, (c) April 2009, and (d) May 2010. We mapped only unequivocal evidence of water vole presence (i.e., food caches or latrines). We did not survey side streams. Main releases of water voles took place in the northern half of the river during August 2006, and in the southern half of the river during August 2007.

After releases in 2007, water vole distribution remained fairly constant through to 2010 (Fig. 6). In April 2010, water voles were distributed along 13.3 km of the River Dore.

## DISCUSSION

Although opportunities to detect mink were systematically maintained for 4 years throughout the treatment area, detections showed a long-term decline from the start, becoming less frequent both spatially and temporally. This overall decline occurred despite progressive expansions of the treatment area, which in some river sections led to a temporary increase in detections and captures, which we took to be the population resident in each section. Following this clearance phase in each river section, detections, and captures showed a seasonal pattern with peaks in spring and autumn coinciding with known periods of population movement. New mink detections typically appeared simultaneously at strings of adjacent rafts, and ceased following removal of 1 or a few mink. We found no evidence of young being reared to independence within the treatment area. From mapped data (see Game & Wildlife Conservation Trust 2012), we identified at least 4 areas of the catchment where mink appeared suddenly during mating and dispersal periods, suggesting routes of entry. Three of these suggested travel from neighboring catchments across watersheds where the height of land was only 5–75 m above the nearest part of the Monnow river system. We estimated response time to be on average less than 13 days, indicating that incoming mink were present in the treatment area for no longer than this before being caught and euthanized.

In summary, detection history suggests that mink presence in the treatment area was brought to, and held at, near-zero levels by the trapping strategy and that after the first year immigration from outside the treatment area determined detections and captures. This seems to contradict the conclusion by King et al. (2009) that invasive Mustelids were difficult to control because of intrinsic behaviors that limited their trapability. These authors showed that even the combined use of passive recorders, traps and lethal bait dispensers could fail to detect ferrets (i.e., feral domesticated *Mustela putorius*) that had been previously caught and tagged and were known still to be alive in the area. They found evidence for inadequate detector density, too brief a detection period, seasonal differences in detectability, and trap- or bait-shyness (including avoidance of infra-red illumination in camera traps). Some of these issues might be attributed to the ferrets' earlier experience of traps, but they highlight the need for caution in interpreting naïve occupancy data (i.e., without correction for uncertain detectability).

The raft-guided strategy used in the present study avoids most of the hazards identified by King et al. (2009). Rafts are located along a linear habitat which is known to be strongly favored by mink. Raft spacing has been pre-determined to promote multiple detections of each mink present (Reynolds et al. 2010, Porteus et al. 2012). Rafts do not carry prominent scent cues because the clay-and-sand medium is a natural material, and no bait or scent lure is used. The raft is also washed clean at each inspection. Thus detection is not expected to be aversive, and indeed mink clearly re-visit rafts in monitoring mode and are readily captured when a trap is set on the same rafts. Capture occurs only once, so mink have no opportunity to learn trap-shyness unless they observe other captures. Even the latter circumstance may not have been aversive: on a few occasions in the present study, we caught several mink on consecutive days on the same raft or adjacent rafts.

Nevertheless, for any species with low detectability, it is difficult to find a measure of population control success that is independent of the control method, does not involve similar cues that might cause avoidance, and has comparable sensitivity. For mink, rafts are the best detector we have; for instance, during 6,411 man-hours of field work in this project, no mink were seen by fieldworkers except in traps. In the raft-guided strategy, detection and capture are not independent, with the result that detectability and occupancy are confounded. Proving the absence of un-detectable mink within the trapped area is impossible, so we must use a probabilistic argument, requiring estimates of detectability and trappability on rafts.

During the 4-year study, we did not detect mink on any raft in the treatment area during 42/166 check periods (25%), forming long periods without detections during both spring and summer (up to 9 consecutive weeks with no mink detected in year 4) and winter (up to 5 consecutive weeks). Given the detectability implied by earlier work with rafts (Reynolds et al. 2010, Porteus et al. 2012) and by capture rates as calculated in this study, the probability of detection failure for such extended periods would be very small indeed. The probability of failing to detect 1 mink for 5 weeks given even 1 raft within its activity range would be expected to be around (1 − 0.059)^5×7^ = 0.12. However, we apparently detected individual mink at more than 1 site, as intended by the raft spacing. Where non-target confusion could be excluded, we obtained 574 detections for 113 mink captured. This implies either that many mink remained uncaught and had dramatically variable detectability, or that each mink caught was detected 5 times. If we accept the latter case as more plausible, the probability of detecting each mink would be >0.95 after only 1.4 weeks of monitoring. The risk of failing to detect it at 5 rafts for 5 weeks would be <0.00004.

Capture of mink following detections was usually rapid, giving no suggestion of trap avoidance among those mink detected. Although we apparently detected mink multiple times before capture, they would presumably have been detected even more times if capture had not followed so rapidly. Less than 2% (8/574) of detections (typically at single rafts, rather than on strings of adjacent rafts) were not followed by any obviously related mink capture (see Game & Wildlife Conservation Trust 2012). These few cases potentially represent 1) mis-identification of non-target tracks as mink, which we have shown to be a low risk; 2) drastic variations in detectability; 3) mink that died of other causes before they could be trapped; and 4) transient mink that left the study area before they could be trapped. The latter 2 categories could have inflated the apparent capture rate by right-censoring capture histories, but would have had at most a small effect because of the small number of rafts involved. Isolated detections at widely spaced rafts that were not followed by captures or repeated at subsequent checks, suggested fast-moving mink that left the catchment. This pattern occurred particularly in spring (e.g., check period 70; see Game & Wildlife Conservation Trust 2012), and we speculate that it characterized roving males seeking females. Seasonal peaks in mink detections led to corresponding peaks in captures. Nineteen percent of captures occurred during the mating season and 64% during the autumn dispersal period. Because we did not use bait or other attractants on rafts or in traps, we do not expect enhanced detectability in these seasons as a result of sex or hunger cues.

From a conservation perspective, the ultimate measure of effectiveness is whether vulnerable prey species benefited. The fate of native and reintroduced water vole colonies has been found to be extremely sensitive to the presence of mink even where habitat is good (Barreto et al. 1998, Bonesi et al. 2007, Harrington et al. 2009, Moorhouse et al. 2009). On the basis of our earlier studies, we were confident that we could quickly control mink density on the Monnow, and that simultaneous water vole reintroduction would therefore be feasible.

The potential local conservation legacy and wider demonstration value undoubtedly made the project easier to fund. However, this was not a replicated experiment to quantify the contribution of mink predation to the risk of water vole extinction. Many factors besides predation could cause a failed reintroduction (e.g., small founder population size, sub-optimal habitat, regular flooding, and native predators). Control of mink predation may a necessary condition for water vole persistence but it is not a sufficient one. Hence, regardless of the fate of this water vole population, our conclusion—based on mink occupancy data—would still have been that trapping held mink at near-zero levels. Nevertheless the establishment and persistence of water voles along the River Dore, despite the other hazards, contrasts with the fate of water voles where mink have not been effectively controlled.

## MANAGEMENT IMPLICATIONS

We demonstrated that a focused trapping strategy based on the use of rafts to solicit mink detections, with traps used only where detections occurred, provided a sufficiently fast response to render a sizeable catchment free of mink and to maintain this situation in the face of repeated immigration. This study was particularly valuable because by using paid professional staff exclusively, a luxury not available in many conservation contexts, it provided consistent effort and detailed monitoring data to demonstrate the impact of trapping. We showed that rapid response to reinvasion was critical to maintaining near-zero levels of mink, thus emphasizing the importance of sustained monitoring effort. By simultaneously re-introducing a native prey species endangered by the presence of alien mink, we demonstrated the utility of systematic trapping as a viable wildlife management tool in species conservation.
